# Dissociation and Culture of Adult Mouse Satellite Glial Cells

**DOI:** 10.21769/BioProtoc.4906

**Published:** 2023-12-20

**Authors:** Raquel Tonello, Steve Davidson, Temugin Berta

**Affiliations:** 1 Pain Research Center, Department of Anesthesiology, University of Cincinnati Medical Center, Cincinnati 45267, USA; 2 Pain Research Center, Department of Molecular Pathobiology, College of Dentistry, New York, USA

**Keywords:** Satellite glial cells, Glial cells, Dorsal root ganglia, Pain, Chronic pain, Cell culture, Quantitative real-time RT-PCR, Calcium imaging

## Abstract

Satellite glial cells (SGCs) are a type of glial cell population that originates from neural crest cells. They ultimately migrate to surround the cell bodies of neurons in the ganglia of the peripheral nervous system. Under physiological conditions, SGCs perform homeostatic functions by modifying the microenvironment around nearby neurons and provide nutrients, structure, and protection. In recent years, they have gained considerable attention due to their involvement in peripheral nerve regeneration and pain. Although methods for culturing neonatal or rat SGCs have long existed, a well-characterized method for dissociating and culturing adult SGCs from mouse tissues has been lacking until recently. This has impeded further studies of their function and the testing of new therapeutics. This protocol provides a detailed description of how to obtain primary cultures of adult SGCs from mouse dorsal root ganglia in approximately two weeks with over 90% cell purity. We also demonstrate cell purity of these cultures using quantitative real-time RT-PCR and their functional integrity using calcium imaging.

Key features

• Detailed and simplified protocol to dissociate and culture primary satellite glial cells (SGCs) from adult mice.

• Cells are dissociated in approximately 2–3 h and cultured for approximately two weeks.

• These SGC cultures allow both molecular and functional studies.

**Graphical overview**


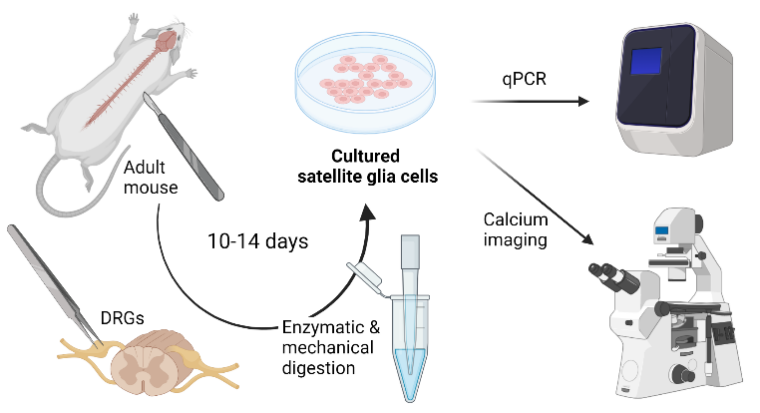


**Dissociation and culture of mouse satellite glial cells**

## Background

Satellite glial cells (SGCs) are a major cellular component of the peripheral nervous system, surrounding the cell bodies of sensory neurons in dorsal root (DRG) and trigeminal ganglia (Hanani, 2005). Long thought to be merely supportive of neurons, increasing evidence suggests that SGCs are dynamic regulators of neuronal processing, including pain on the peripheral nervous system (Ji et al., 2013). Pain affects more than 1.5 billion people worldwide, with hundreds of millions suffering from unrelieved chronic pain (Kennedy et al., 2014). Given the overuse of opioid prescriptions to treat chronic pain, new therapeutics are urgently needed (Grosser et al., 2017).

While there is a growing consensus that SGCs play a role in important physiological and pathological pain mechanisms (Hanani and Spray, 2020; Gazerani, 2021; Andreeva et al., 2022) and may provide new therapeutic targets for pain relief, our knowledge of SGC biology is still limited because of the lack of techniques to study these cells in vitro. Previous protocols for the dissociation and culture of SGCs have been generated from neonatal and immature tissues, contained neuronal and non-neuronal cells, or mostly used rats (Chen et al., 2008; Belzer et al., 2010; de Corato et al., 2011; Poulsen et al., 2014; Wang et al., 2019). Recently, there have been publications using cultured SGCs from adult mice (Su et al., 2022; Tonello et al., 2023). However, a detailed and well-characterized protocol for the dissociation and culture of mouse SGCs is still lacking.

Here, we describe a step-by-step protocol used in our recent publication (Tonello et al., 2023) that dissociates adult SGCs from mouse DRG tissues. The protocol results in cultures that are more than 90% pure and take approximately 10–14 days to be suitable for molecular and functional testing. As a proof of concept, we have also characterized these cultures for the transcriptional expression of SGC-enriched genes, such as the endothelin receptor type B (EDNRB), and functional responses to a specific EDNRB agonist by calcium imaging.

## Materials and reagents

**Biological materials**

1. CD1 mouse (6–12 weeks) (Charles River, catalog number: 022)

**Reagents**

1. Hanks’ balanced salt solution (HBSS) without Ca^2+^/Mg^2+^ (Thermo Fisher Scientific, catalog number: 14175095)
2. 1 M HEPES (Thermo Fisher Scientific, catalog number: 15630080)
3. Penicillin-Streptomycin (P/S), 10,000 U/mL (Thermo Fisher Scientific, catalog number: 15140122)
4. Papain (buffered aqueous suspension, ≥16 units/mg protein) (Millipore Sigma, catalog number: P3125)
5. Collagenase from *Clostridium histolyticum* (Millipore Sigma, catalog number: C6885)
6. Dulbecco’s modified Eagle medium (DMEM), low glucose (Thermo Fisher Scientific, catalog number: 11885084)
7. Fetal bovine serum (FBS), heat inactivated (Fisher Scientific, catalog number: MT35011CV)
8. Amphotericin B (Thermo Fisher Scientific, catalog number: 15290026)

**Solutions**

*Note: Prepare all solutions in a laminar flow cell culture hood.*

1. HBSS dissociation solution (see Recipes)
2. Papain solution (see Recipes)
3. Collagenase stock (see Recipes)
4. Collagenase solution (see Recipes)
5. DMEM culture medium (see Recipes)

**Recipes**

1. **HBSS dissociation solution (store at 4 °C for up to six months)**
  **Table:** 

Reagent	Final concentration	Quantity
HBSS	n/a	490 mL
HEPES	1% (v/v)	5 mL
P/S	1% (v/v)	5 mL
Total	n/a	500 mL
2. **Papain solution (freshly prepared and used)**
  **Table:** 

Reagent	Final concentration	Quantity
Papain	40 units total	108 μL
HBSS dissociation solution	n/a	3 mL
Total	n/a	3.108 mL
3. **Collagenase stock (aliquot at 200 μL, and store at -20 °C for up to six months)**
  **Table:** 

Reagent	Final concentration	Quantity
Collagenase	22.5 mg/mL	100 mg
HEPES	1% (v/v)	44 μL
HBSS	n/a	4.4 mL
Total	n/a	4.444 mL
4. **Collagenase solution (freshly prepared and used)**
  **Table:** 

Reagent	Final concentration	Quantity
Collagenase stock	1.5 mg/mL	200 μL
HBSS dissociation solution	n/a	2.8 mL
Total	n/a	3.0 mL
5. **DMEM culture medium (store at 4 °C for up to six months)**
  **Table:** 

Reagent	Final concentration	Quantity
FBS	10% (v/v)	50 mL
P/S	1% (v/v)	5 mL
Amphotericin B	1% (v/v)	5 mL
DMEM, low glucose	n/a	440 mL
Total	n/a	500 mL

**Laboratory supplies**

1. Isoflurane (Henry Schein, catalog number: 1182097)
2. Spray bottle with 70% ethanol (Fisher Scientific, catalog number: BP82031GAL)
3. Tissue culture dish (35 × 10 mm) (Fisher Scientific, catalog number: 08-772A)
4. Microtubes, 1.7 mL (Fisher Scientific, catalog number: 14-222-168)
5. Centrifugation tubes, 50 mL (Fisher Scientific, catalog number: 06-443-18)
6. Cell strainer 40 μm (Fisher Scientific, catalog number: 22-363-547)
7. Cell strainer 10 μm (PluriSelect, catalog number: 43-50010-03)
8. Cell culture 12-well microplates (Fisher Scientific, catalog number: 07-000-202)
9. Cover glass (Fisher Scientific, catalog number: 22-050-232)

## Equipment

1. Stereomicroscope system (Olympus, catalog number: SZ51)
2. Biosafety cabinet (Fisher Scientific, catalog number: NC0986267)
3. Isotemp water bath (Fisher Scientific, catalog number: FSGPD2S)
4. Water-jacketed CO_2_ incubator (Fisher Scientific, catalog number: 13-998-078)
5. Sorvall Legend Micro 21R microcentrifuge (Thermo Fisher Scientific, catalog number: 75002445)
6. Centrifuge 5702 (Eppendorf, catalog number: 022628102)
7. Standard scissors (Fine Science Tools, catalog number: 14002-12)
8. Forceps (World Precision Instruments, catalog number: 501987)
9. Student spring scissors (Fine Science Tools, catalog number: 91500-09)
10. Dumont #5 forceps (Fine Science Tools, catalog number: 11251-20)
11. Gilson (or equivalent) pipettes and tips (P20/P200/P1000) (Fisher Scientific, catalog number: various)

## Procedure

**Figure 1.**
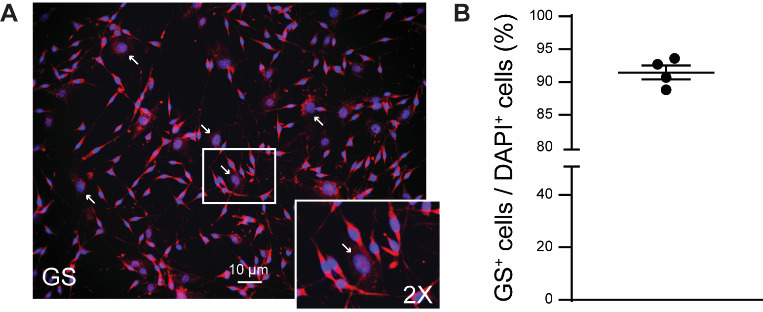
Cell purity of satellite glial cell (SGC) cultures. A. Representative image of immunofluorescence of GS protein and DAPI in SGC culture after 10–14 days. Arrows indicate large-nuclei cells (e.g., fibroblasts). B. Percentage of GS+ cells (i.e., SGCs) over DAPI + cells (i.e., all cells). Graph was generated using GraphPad Prism (v. 10.0.2). Mean ± SEM, n = 4 independent SGC cultures.

- **Initial preparations**
  1. Prior to beginning any procedures, disinfect all surgical tools and working areas with 70% ethanol.
  2. Next, prepare a culture dish by filling it with 4 mL of HBSS dissociation solution (see Recipes) for the initial collection and cleaning of DRG tissues. Prepare two microtubes with 1 mL of HBSS dissociation solution each, where the cleaned tissues will be transferred. Place the culture dish and microtubes on ice at 4 °C.
  3. The papain and collagenase solutions (see Recipes) can also be prepared at this time and kept at 4 °C. Warm these solutions and DMEM culture medium (see Recipes) to 37 °C just before use.
  4. Terminally anesthetize a mouse by placing the animal in a jar/chamber for anesthesia. Add a gauze with 1 mL of isoflurane to the jar, and the mouse will be anesthetized within a minute. To ensure complete anesthesia, check for a lack of response to a pinch of the rear footpad.
  5. Next, use scissors to decapitate the mice in a sink for the purpose of euthanasia and blood removal. This will facilitate the tissue dissection process.
- **Tissue dissection**
  1. Move the mouse to a designed tissue dissection area and liberally spray the back fur with 70% ethanol.
  2. To expose the back muscles, make a large transverse cut in the middle of the back skin using standard scissors. Then, pull the skin in opposite directions to remove all of the back skin.
  3. To remove the back muscles, make two long cuts close to the left and right sides of the spinal column. Then, use forceps to pull away the muscles starting from the rostral end.
  4. To expose the spinal cord and DRGs, perform a laminectomy by removing the top of the vertebral canal. Use spring scissors to cut the bones on both sides of the vertebral canal in a 45° angle to avoid damaging or losing the DRGs. Continue cutting from side to side while lifting the top of the vertebral canal, starting from the rostral end and working your way down to the caudal end of the spinal column.
  5. Next, use forceps to slowly remove the spinal cord in a rostral to caudal direction from the column and expose the DRGs. Discard the spinal cord. The DRGs are located in the dorso-lateral position of the vertebral canal and can be recognized by their round shape and hyaline appearance, which is different from the white color of the attached nerve fibers (Supplementary Figure 1).
  6. Using the stereomicroscope, grasp the dorsal root with #5 forceps, carefully pull it a few millimeters, and cut the spinal nerve (immediately distal to the ganglion) with spring scissors. Next, cut the dorsal root and place the collected DRGs in the culture dish on ice. Typically, an experienced experimenter can collect approximately 40 ganglia per mouse.
  7. If needed, move the culture dish under the stereomicroscope and use a pair of spring scissors and #5 forceps to remove any extra nerves, roots, and connective tissues from the DRGs. Then, transfer the cleaned DRGs equally into two microtubes.
- **Cell dissociation**
  1. Centrifuge the microtubes containing the cleaned DRGs at 200× *g* for 1 min and remove the HBSS dissociation solution.
  2. Perform the first enzymatic dissociation by adding 1.5 mL of papain solution into the microtubes. Gently flick the microtubes to resuspend the DRGs and incubate them for 20 min in a 37 °C water bath.
*Note: Do not resuspend with a pipette or vigorously shake to maintain the integrity of the cells.*
  3. Centrifuge the microtubes at 200× *g* for 1 min and remove the papain solution.
  4. Wash the DRGs with 1 mL of HBSS dissociation solution, centrifuge (200× *g* for 1 min), and remove the solution.
  5. Perform the second enzymatic dissociation by adding 1.5 mL of collagenase solution into the microtubes. Gently flick the microtubes to resuspend the DRGs and incubate them for 20 min in a 37 °C still water bath.
*Note: Do not resuspend with the pipette or vigorously shake to maintain the integrity of the cells.*
  6. Centrifuge the microtubes at 200× *g* for 1 min and remove all the collagenase solution.
*Note: From this point forward, the opening of tubes/plates that contain any tissue, cells, media, or reagents should be done in a laminar flow cell culture hood.*
  7. Resuspend the DRGs in 1 mL of DMEM culture medium. Centrifuge at 200× *g* for 1 min and carefully remove all culture solution without disturbing the tissue.
  8. Resuspend the DRGs in 0.5 mL of DMEM culture medium. Perform mechanical dissociation by trituration using a P1000 pipette until the solution becomes cloudy (approximately 15–20 times up and down). During trituration, the tissues should pass through the tip with a little friction initially and progress to passing easily. Be gentle and avoid introducing air bubbles at this step; this is critical.
  9. Add 1 mL of DMEM culture medium to each microtube. Next, transfer the contents of all microtubes into one 50 mL conical tube. Adjust the volume to approximately 5 mL with DMEM culture medium and gently mix the contents by flicking the tube.
  10. Filter the mixture using a 40 μm cell strainer followed by a 10 μm cell strainer. Collect the filtrate each time in a 50 mL tube. If necessary, adjust the volume of the filtrate to 5 mL with DMEM culture medium and resuspend the cells by gently flicking the tube.
- **Cell plating and growth**
  1. Transfer the resuspended cells into a 12-well microplate by pipetting 400 μL of the filtrate into each well containing sterilized cover glasses. Then, incubate the plate in a CO_2_ cell culture incubator (37 °C, 5% CO_2_).
*Note: During this incubation (24 h), due to the lack of coating of the cover glasses and use of DMEM low-glucose media, SGC cells will attach to the bottom of the dish while the DRG neurons and debris will remain in suspension.*
  2. One day after plating the cells, replace the DMEM culture medium with 400 μL of fresh DMEM culture medium to remove cell debris. Always pre-warm the culture medium to 37 °C before use.
  3. SGCs proliferate and develop a spindle-shaped morphology (Figure 1) during the next 10–14 days. To maintain SGCs cultures, replace the DMEM culture medium every 2–3 days.

## Validation of protocol

In our research article, we used this protocol to study the role of matrix metalloproteinases in SGCs and pain (Tonello et al., 2023). Here, we present additional evidence that this protocol is both robust and reproducible by assessing these cultures using molecular and functional approaches.

**SGC cultures with an average cell purity of over 90%**

We assessed the purity of cell culture using immunofluorescence, following the method described in our publication (Tonello et al., 2023). In brief, after 10–14 days, we replaced the DMEM culture medium with a 4% paraformaldehyde solution and incubated it for 15 min at room temperature (RT). Next, we rinsed the cultures with PBS and blocked them with 1% BSA and 0.2% Triton X-100 in PBS (BSA solution). We then incubated them overnight at 4 °C in the BSA solution with the anti-glutamine synthetase primary antibody (GS, rabbit, 1:5,000). Afterward, we incubated the cultures with the anti-rabbit secondary antibodies conjugated to Alexa Fluor 546 (1:1,000) for 1 h at RT in the BSA solution. Finally, we treated them with the universal nuclear staining DAPI in PBS for 1 min at RT. We then removed the cover glasses from the microplate and covered the side with the cultured SGCs with Prolong Gold Antifade Mountant, mounting them on glass microscope slides. Images were acquired using a Keyence BZ-X800 microscope, and cells were manually counted using NIH Image J open-source software (Schindelin et al., 2012). Cell purity was assessed as a percentage, calculated by dividing the number of GS+ cells (i.e., SGCs) by the number of DAPI+ cells (i.e., all cells in the culture) (Figure 1A). Our protocol resulted in SGC cultures with an average cell purity of 91.4% ± 1.1% (Figure 1B). Approximately 9% of the cells positive only for DAPI were considered potential contaminants. Fibroblasts, characterized as flat cells with a large nucleus, were identified as a potential major contaminant (Figure 1A). It should be noted that a reduced expression of GS has been reported in SGC culture (Belzer et al., 2010). Other markers of SGC, such as fatty acid binding protein 7 (Avraham et al., 2020), may be used instead.

**Cultures show high expression of SGC transcriptional markers**

**Figure 2.**
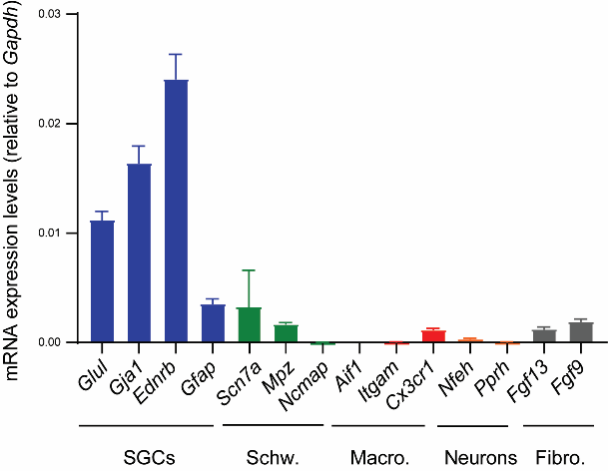
Transcriptional analysis of satellite glial cell (SCG) cultures. Quantification of various cell markers using qPCR shows the higher expression of markers for SGCs over markers for Schwann cells (Schw.), macrophages (Macro.), neurons, and fibroblasts (Fibro.). The graph was generated using GraphPad Prism (v. 10.0.2). Mean ± SEM, n = 6 independent SGC cultures.

We assessed the transcriptional expression levels of various cell markers using qPCR, following the method described in our publication (Tonello et al., 2023). In brief, after 10–14 days, we replaced the DMEM culture medium with TRIzol reagent to lyse SGCs and isolate the RNA. We extracted the total RNA from the TRIzol reagent using the Direct-zol RNA MiniPrep kit and then assessed the amount and quality using a UV-Vis spectrophotometer. We then converted the RNA into cDNA using a high-capacity cDNA reverse transcription kit. Specific primers for various cell markers and *Gapdh* (used for normalization) were obtained from PrimerBank (Wang et al., 2012). The primer sequences can be found in Supplementary Table 1. qPCR was conducted using PowerUp SYBR Green Master Mix on a QuantStudio 3 Real-Time PCR System. Relative transcriptional expression ratios were calculated using the Pfaffl method (Pfaffl, 2001). Figure 2 shows that our protocol resulted in cultures with high expression of SGC gene markers such as *Glul, Gja1*, and *Ednrb* (Feldman-Goriachnik and Hanani, 2017; Hanani and Spray, 2020), while showing low expression of gene markers for non-myelinated (*Scn7a*) and myelinated Schwann cells (*Mpz* and *Ncmap*), macrophages (*Aif1, Itgam*, and *Cx3xr1*), neurons (*Nefh* and *Prph*), and fibroblasts (*Fgf13* and *Fgf9*) (Avraham et al., 2020; Jager et al., 2020; Tonello et al., 2020; Chu et al., 2023; Tonello et al., 2023).

**Cultures exhibit functional response to EDNRB agonist**

**Figure 3.**
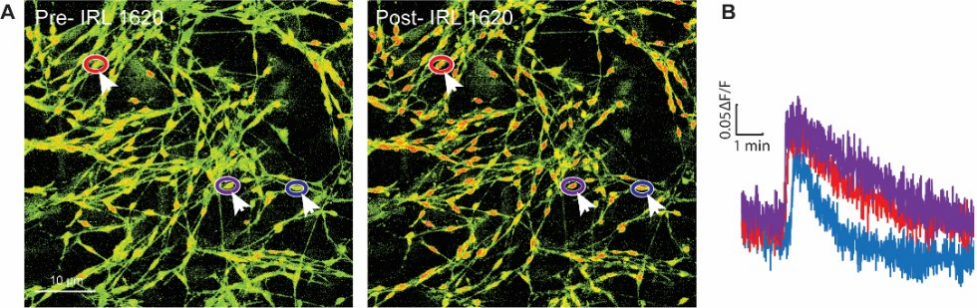
Calcium responses in satellite glial cell (SGC) cultures. (A) Images of Fura-2 signaling in SGC cultures and (B) representative calcium imaging traces of SGCs (arrows) in response to the EDNRB agonist IRL 1620 (10 nM). Colors correspond to encircled regions of interest in A.

We assessed the functional integrity of our SGC cultures using calcium imaging, following the method outlined in our publications (Lee et al., 2020; Ford et al., 2021). In brief, after 10–14 days, we replaced the DMEM culture medium and incubated the SGC culture for 45 min at room temperature with a solution of Fura2-AM (3 μg/mL) in DMEM containing 10% FBS and 1% P/S. We then removed the cover glasses from the cell culture microplates and transferred them to a recording chamber containing an external recording solution with the following concentrations: 130 mM NaCl, 5 mM KCl, 2 mM CaCl_2_, 1 mM MgCl_2_, 30 mM glucose, and 10 mM HEPES. Illuminance signal was acquired using 365/385-nm switching LED controlled by the MetaFluor software (Molecular Devices) on an Olympus BX51 microscope. Previous research shows that endothelin-1 effectively increases intracellular calcium levels in SGCs by activating the endothelin receptor EDNRB (Feldman-Goriachnik and Hanani, 2017). Here, we show that IRL 1620 (10 nM), a potent and selective EDNRB agonist (Takai et al., 1992), induced an intracellular calcium increase in most, if not all, SGCs (Figure 3A and B). This experiment serves as a proof of concept for the functional integrity of our SGC cultures. These cultures may be used in future studies to evaluate and measure calcium responses to various agonists, including neurotransmitters or cytokines (Souza et al., 2013; Afroz et al., 2019).

## General notes and troubleshooting

SGCs have been identified as a potential therapeutic target for the treatment of chronic pain. However, our understanding of SGC biology is limited due to the lack of techniques to study these cells in vitro. Here, we provide a step-by-step protocol for the dissociation and culture of adult mouse SGCs:

1. This protocol is easy to implement and takes approximately 2 h for one person to complete the entire dissection and plating process with some practice. Typically, CD1 mice aged 8–12 weeks are used for this protocol. However, this protocol has been successful with mice of different ages and genetic backgrounds.
2. Dissection of DRG tissues is carried out similarly to a previously published protocol for the production of sensory neurons (Malin et al., 2007). However, we take extra care to remove any attached spinal nerve and dorsal root to minimize potential contamination from Schwann cells (Supplementary Figure 1).
3. To maximize cell survival and number in culture, we recommend a dissection time of no more than 30–45 min. Tissues should never be left on ice for longer than 1 h.
4. The growth of SGC cultures on cover glasses is not necessary. Dissociated cells can be plated directly into a 12-well microplate, for example for qPCR analysis.
5. Cultured SGCs require 10–14 days of incubation to reach a confluency of 60%–80% and acquire a spindle-shaped, bipolar morphology. Longer incubations risk contamination by fibroblasts.
  In conclusion, this protocol results in cultures more than 90% pure and suitable for molecular and functional testing. These cultures may be used to better understand the biology of SGCs and to develop new therapeutics for the relief of chronic pain.

## Supplementary information

The following supporting information can be downloaded here:

1. Supplementary Figure 1
2. Supplementary Table 1
